# *catena*-Poly[[silver(I)-μ-*N*-(pyridin-3-ylmeth­yl)pyridine-2-amine-κ^2^ *N*:*N*′] tri­fluoro­methane­sulfonate]

**DOI:** 10.1107/S1600536813016309

**Published:** 2013-06-26

**Authors:** Suk-Hee Moon, Ki-Min Park

**Affiliations:** a Department of Food & Nutrition, Kyungnam College of Information and Technology, Busan 617-701, Republic of Korea; b Department of Chemistry and Research Institute of Natural Sciences, Gyeongsang National University, Jinju 660-701, Republic of Korea

## Abstract

In the asymmetric unit of the title polymeric complex, {[Ag(C_11_H_11_N_3_)](CF_3_SO_3_)}_*n*_, there are two Ag^I^ atoms, two *N*-(pyridin-3-ylmeth­yl)pyridine-2-amine ligands (*A* and *B*) and two CF_3_SO_3_^−^ anions. One Ag^I^ atom is coordinated by two pyridine N atoms from two symmetry-related *A* ligands in a geometry slightly distorted from linear [N—Ag—N = 173.2 (3)°], forming a left-handed helical chain, while the other Ag^I^ atom is coordinated by two pyridine N atoms from two symmetry-related *B* ligands in a bent arrangement [N—Ag—N = 157.1 (3)°], forming a right-handed helical chain. Both helical chains have the same pitch length [10.4007 (7) Å], propagate along the *b-*axis direction and are alternately arranged *via* Ag⋯Ag [3.0897 (12) Å] and π–π stacking inter­actions [centroid–centroid distances = 3.564 (7) and 3.518 (6) Å], resulting in the formation of a two-dimensional supra­molecular network extending parallel to the *ab* plane. Inter­molecular N—H⋯O, C—H⋯O and C—H⋯F hydrogen-bonding inter­actions occur between the helical chains and the anions.

## Related literature

For related structures and applications of Ag^I^ coordination polymers with dipyridyl ligands, see: Leong & Vittal (2011 ▶); Moulton & Zaworotko (2001 ▶). For the crystal structure of the related perchlorate salt, see: Zhang *et al.* (2013 ▶). For the synthesis of the ligand, see: Foxon *et al.* (2002 ▶); Lee *et al.* (2008 ▶).
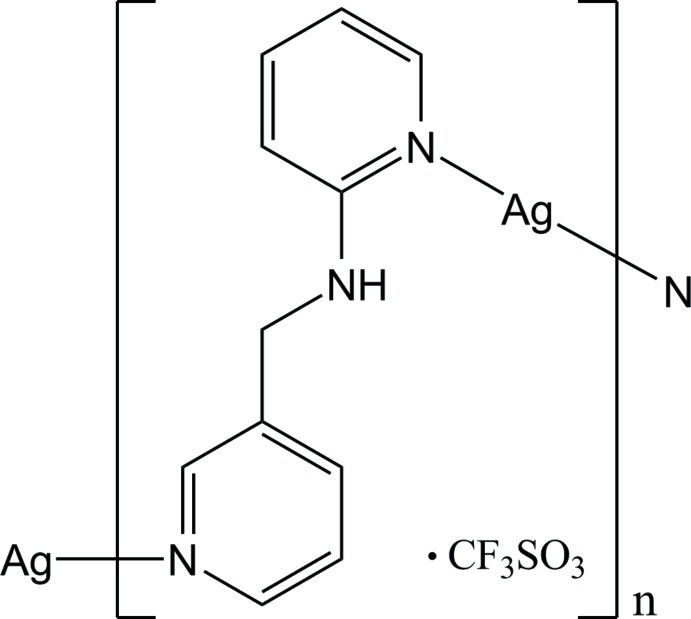


## Experimental

### 

#### Crystal data

[Ag(C_11_H_11_N_3_)](CF_3_O_3_S)
*M* *_r_* = 442.17
Monoclinic, 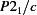

*a* = 14.0965 (10) Å
*b* = 10.4007 (7) Å
*c* = 20.6593 (15) Å
β = 102.994 (1)°
*V* = 2951.4 (4) Å^3^
*Z* = 8
Mo *K*α radiation
μ = 1.56 mm^−1^
*T* = 173 K
0.30 × 0.25 × 0.25 mm

#### Data collection

Bruker SMART CCD area-detector diffractometer
Absorption correction: multi-scan (*SADABS*; Bruker, 2000 ▶) *T*_min_ = 0.652, *T*_max_ = 0.697
16169 measured reflections
5797 independent reflections
4479 reflections with *I* > 2σ(*I*)
*R*_int_ = 0.047

#### Refinement

*R*[*F* ^2^ > 2σ(*F* ^2^)] = 0.088
*wR*(*F* ^2^) = 0.238
*S* = 1.09
5797 reflections
415 parameters
6 restraints
H-atom parameters constrained
Δρ_max_ = 2.70 e Å^−3^
Δρ_min_ = −1.89 e Å^−3^

### 

Data collection: *SMART* (Bruker, 2000 ▶); cell refinement: *SAINT-Plus* (Bruker, 2000 ▶); data reduction: *SAINT-Plus*; program(s) used to solve structure: *SHELXS97* (Sheldrick, 2008 ▶); program(s) used to refine structure: *SHELXL97* (Sheldrick, 2008 ▶); molecular graphics: *DIAMOND* (Brandenburg, 2005 ▶); software used to prepare material for publication: *SHELXTL* (Sheldrick, 2008 ▶).

## Supplementary Material

Crystal structure: contains datablock(s) I, global. DOI: 10.1107/S1600536813016309/sj5332sup1.cif

Structure factors: contains datablock(s) I. DOI: 10.1107/S1600536813016309/sj5332Isup2.hkl

Additional supplementary materials:  crystallographic information; 3D view; checkCIF report

## Figures and Tables

**Table 1:** Hydrogen-bond geometry (Å, °)

*D*—H⋯*A*	*D*—H	H⋯*A*	*D*⋯*A*	*D*—H⋯*A*
N3—H3⋯O4	0.88	2.20	3.021 (12)	156
N6—H6⋯O2^i^	0.88	2.42	3.159 (12)	142
C1—H1⋯O1	0.95	2.56	3.389 (16)	146
C6—H6*A*⋯F6^ii^	0.99	2.55	3.282 (15)	131
C9—H9⋯O3^iii^	0.95	2.44	3.329 (14)	156
C10—H10⋯O1^iv^	0.95	2.57	3.373 (14)	142
C12—H12⋯O4^v^	0.95	2.51	3.331 (15)	145
C17—H17*A*⋯O3^i^	0.99	2.42	3.186 (14)	134
C21—H21⋯F6^vi^	0.95	2.54	3.325 (14)	140

Symmetry codes: (i) 
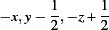
; (ii) 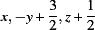; (iii) 
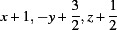
; (iv) 
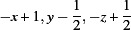
; (v) 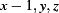; (vi) 
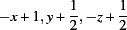
.

**Fig. 1.**
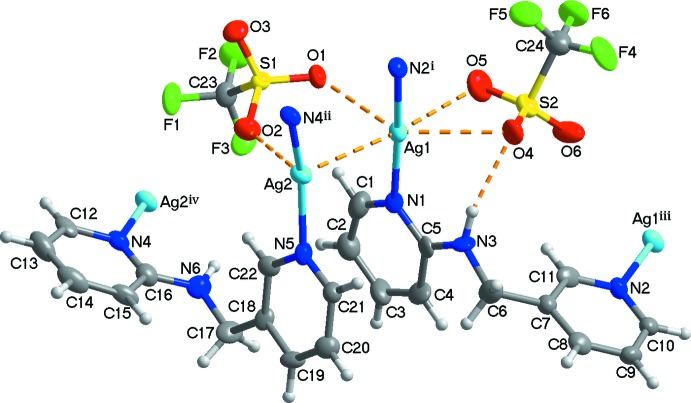
A view of the molecular structure of the title compound, with atom numbering. Displacement ellipsoids are drawn at the 50% probability level and dashed lines present Ag···O and N–H···O contacts. [Symmetry codes: (i) 1 - x, 1/2 + y, 1/2 - z; (ii) -x, 1/2 + y, 1/2 - z; (iii) 1 - x, -1/2 + y, 1/2 - z; (iv) -x, -1/2 + y, 1/2 - z].

**Fig. 2.**
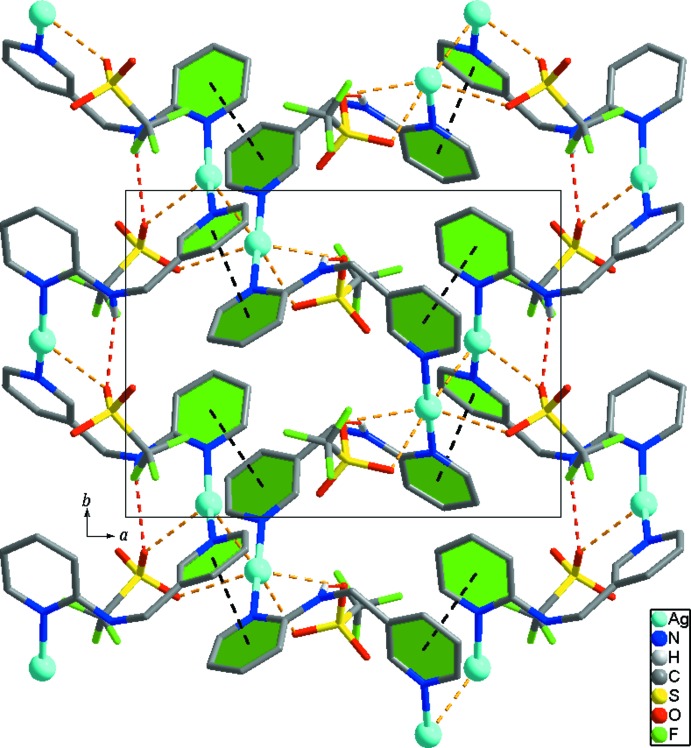
The two-dimensional supramolecular structure formed through Ag···Ag and Ag···O interactions (yellow dashed lines) and π–π stacking interactions (black dashed lines). Red dashed lines present N–H···O hydrogen bonds.

## supplementary crystallographic information

### Comment

Silver coordination polymers based on dipyridyl type ligands have attracted particular interest because of the intriguing architectures caused by a variety of coordination geometries for the Ag(I) cation as well as their potential applications as functional materials (Leong & Vittal, 2011; Moulton & Zaworotko, 2001). However, despite the rapid growth in the Ag(I) coordination chemistry based on symmetrical dipyridyl ligands, investigations using unsymmetrical dipyridyl ligands with nitrogen donor atoms in different positions on the two terminal pyridines still remains lacking. Herein, we report the crystal structure of the title compound prepared by the reaction of silver trifluoromethanesulfonate with the unsymmetrical dipyridyl ligand. The structure of title compound is isostructural with the perchlorate salt (Zhang *et al.*, 2013).

The asymmetric unit of the title compound contains two Ag^I^ atoms (Ag1 and Ag2), two *N*-(pyridin-3-ylmethyl)pyridine-2-amine (Foxon *et al.*, 2002; Lee *et al.*, 2008) ligands (A and B) and two CF_3_SO_3_^-^ anions. The Ag1 atom is coordinated by two pyridine N atoms from two symmetry-related ligand A in a geometry slightly distorted from linear [N–Ag1–N = 173.2 (3)°] to form left-handed helical chain, while the Ag2 atom is coordinated by two pyridine N atoms from two symmetry-related ligand B in a bent arrangement [N–Ag2–N = 157.1 (3)°] to form right-handed helical chain (Fig. 1). Two pyridine rings coordinated to the Ag1 and Ag2 centers are tilted by 14.2 (7)° and 34.1 (5)°, respectively, with respect to each other. Both helical chains with the same pitch length of 10.4007 (7) Å propagate along the *b* axis and are alternately arranged *via* the Ag···Ag interactions [3.0897 (12) Å], resulting in the formation of a two-dimensional supramolecular network extending parallel to the *ab* plane. Furthermore, π–π stacking interactions [centroid-centroid distances = 3.564 (7) and 3.518 (6) Å] between pyridine rings of both helical chains, as shown in Fig. 2, contribute to stabilize the two-dimensional network.

The non-coordinating CF_3_SO_3_^-^ anions participate in N–H···O hydrogen bonding (Table 1, Fig. 2) and Ag···O interactions (Ag1···O4 2.815 (8), Ag1···O5 2.852 (10), Ag1···O1 2.867 (8), Ag2···O2 2.722 (8) Å) (Fig. 1,2). In addition, C–H···O and C–H···F hydrogen bonds (Table 1) between the helical chains and anions are also detected in the crystal.

### Experimental

The ligand (*N*-(pyridin-3-ylmethyl)pyridine-2-amine) was prepared according to a procedure described by Foxon *et al.* (2002). Crystals of the title compound suitable for X-ray analysis were obtained by vapor diffusion of diethyl ether into a DMSO solution of the white precipitate afforded by the reaction of the ligand with silver(I) trifluoromethanesulfonate in a 1:1 molar ratio in methanol.

### Refinement

All H atoms were positioned geometrically and refined using a riding model, with d(C-H) = 0.95 Å for C*sp*^2^-H, 0.88 Å for amine N-H and 0.99 Å for methylene C-H. For all H atoms *U*_iso_(H) = 1.2*U*_eq_(C,N).

### Crystal data

**Table:**

[Ag(C_11_H_11_N_3_)](CF_3_O_3_S)	*F*(000) = 1744
*M**_r_* = 442.17	*D*_x_ = 1.990 Mg m^−^^3^
Monoclinic, *P*2_1_/*c*	Mo *K*α radiation, λ = 0.71073 Å
Hall symbol: -P 2ybc	Cell parameters from 5912 reflections
*a* = 14.0965 (10) Å	θ = 2.2–28.1°
*b* = 10.4007 (7) Å	µ = 1.56 mm^−^^1^
*c* = 20.6593 (15) Å	*T* = 173 K
β = 102.994 (1)°	Block, colourless
*V* = 2951.4 (4) Å^3^	0.30 × 0.25 × 0.25 mm
*Z* = 8	

### Data collection

**Table:**

Bruker SMART CCD area-detector diffractometer	5797 independent reflections
Radiation source: fine-focus sealed tube	4479 reflections with *I* > 2σ(*I*)
Graphite monochromator	*R*_int_ = 0.047
φ and ω scans	θ_max_ = 26.0°, θ_min_ = 1.5°
Absorption correction: multi-scan (*SADABS*; Bruker, 2000)	*h* = −17→14
*T*_min_ = 0.652, *T*_max_ = 0.697	*k* = −12→12
16169 measured reflections	*l* = −15→25

### Refinement

**Table:**

Refinement on *F*^2^	Primary atom site location: structure-invariant direct methods
Least-squares matrix: full	Secondary atom site location: difference Fourier map
*R*[*F*^2^ > 2σ(*F*^2^)] = 0.088	Hydrogen site location: inferred from neighbouring sites
*wR*(*F*^2^) = 0.238	H-atom parameters constrained
*S* = 1.09	*w* = 1/[σ^2^(*F*_o_^2^) + (0.1255*P*)^2^ + 38.027*P*] where *P* = (*F*_o_^2^ + 2*F*_c_^2^)/3
5797 reflections	(Δ/σ)_max_ = 0.001
415 parameters	Δρ_max_ = 2.70 e Å^−^^3^
6 restraints	Δρ_min_ = −1.89 e Å^−^^3^

### Special details

**Table:**

Geometry. All e.s.d.'s (except the e.s.d. in the dihedral angle between two l.s. planes) are estimated using the full covariance matrix. The cell e.s.d.'s are taken into account individually in the estimation of e.s.d.'s in distances, angles and torsion angles; correlations between e.s.d.'s in cell parameters are only used when they are defined by crystal symmetry. An approximate (isotropic) treatment of cell e.s.d.'s is used for estimating e.s.d.'s involving l.s. planes.
Refinement. Refinement of *F*^2^ against ALL reflections. The weighted *R*-factor *wR* and goodness of fit *S* are based on *F*^2^, conventional *R*-factors *R* are based on *F*, with *F* set to zero for negative *F*^2^. The threshold expression of *F*^2^ > σ(*F*^2^) is used only for calculating *R*-factors(gt) *etc*. and is not relevant to the choice of reflections for refinement. *R*-factors based on *F*^2^ are statistically about twice as large as those based on *F*, and *R*- factors based on ALL data will be even larger.

### Fractional atomic coordinates and isotropic or equivalent isotropic displacement parameters (Å^2^)

**Table:**

	*x*	*y*	*z*	*U*_iso_*/*U*_eq_	
Ag1	0.30400 (6)	0.83549 (8)	0.19418 (4)	0.0296 (3)	
Ag2	0.19133 (6)	1.03934 (8)	0.25579 (4)	0.0301 (3)	
N1	0.2935 (6)	0.6974 (8)	0.2706 (4)	0.0255 (19)	
N2	0.6866 (6)	0.4901 (9)	0.3732 (5)	0.029 (2)	
N3	0.4482 (7)	0.7530 (10)	0.3296 (4)	0.037 (2)	
H3	0.4585	0.7873	0.2929	0.045*	
N4	−0.1876 (6)	0.7099 (8)	0.3036 (4)	0.0268 (19)	
N5	0.2011 (6)	0.9267 (9)	0.3454 (5)	0.029 (2)	
N6	−0.0305 (7)	0.6806 (9)	0.3627 (5)	0.031 (2)	
H6	−0.0245	0.6207	0.3338	0.037*	
C1	0.2165 (9)	0.6290 (13)	0.2671 (7)	0.045 (3)	
H1	0.1679	0.6390	0.2273	0.053*	
C2	0.1944 (9)	0.5454 (12)	0.3110 (7)	0.041 (3)	
H2	0.1341	0.5008	0.3038	0.049*	
C3	0.2670 (9)	0.5300 (13)	0.3677 (7)	0.045 (3)	
H3A	0.2579	0.4718	0.4012	0.055*	
C4	0.3531 (10)	0.5987 (12)	0.3761 (6)	0.041 (3)	
H4	0.4029	0.5883	0.4152	0.050*	
C5	0.3656 (8)	0.6842 (10)	0.3259 (5)	0.028 (2)	
C6	0.5209 (9)	0.7732 (12)	0.3911 (6)	0.041 (3)	
H6A	0.4884	0.7677	0.4288	0.050*	
H6B	0.5474	0.8613	0.3908	0.050*	
C7	0.6058 (8)	0.6777 (10)	0.4031 (5)	0.027 (2)	
C8	0.6852 (8)	0.6987 (10)	0.4541 (6)	0.030 (2)	
H8	0.6856	0.7712	0.4821	0.036*	
C9	0.7637 (8)	0.6182 (11)	0.4657 (5)	0.031 (2)	
H9	0.8177	0.6327	0.5017	0.038*	
C10	0.7620 (8)	0.5156 (11)	0.4235 (5)	0.028 (2)	
H10	0.8170	0.4603	0.4305	0.034*	
C11	0.6099 (8)	0.5723 (11)	0.3626 (5)	0.030 (2)	
H11	0.5568	0.5571	0.3260	0.036*	
C12	−0.2713 (9)	0.7802 (10)	0.2889 (6)	0.031 (2)	
H12	−0.3248	0.7489	0.2562	0.037*	
C13	−0.2807 (10)	0.8951 (12)	0.3200 (7)	0.044 (3)	
H13	−0.3400	0.9421	0.3097	0.053*	
C14	−0.2031 (10)	0.9394 (11)	0.3658 (6)	0.042 (3)	
H14	−0.2082	1.0195	0.3868	0.051*	
C15	−0.1179 (9)	0.8727 (10)	0.3827 (5)	0.030 (3)	
H15	−0.0641	0.9046	0.4151	0.036*	
C16	−0.1127 (8)	0.7554 (10)	0.3505 (6)	0.030 (2)	
C17	0.0462 (9)	0.6965 (11)	0.4212 (6)	0.035 (3)	
H17A	0.0770	0.6118	0.4336	0.041*	
H17B	0.0171	0.7252	0.4581	0.041*	
C18	0.1260 (7)	0.7931 (9)	0.4138 (5)	0.024 (2)	
C19	0.1999 (8)	0.8217 (10)	0.4677 (5)	0.028 (2)	
H19	0.1992	0.7871	0.5102	0.034*	
C20	0.2750 (9)	0.9011 (11)	0.4598 (6)	0.034 (3)	
H20	0.3263	0.9219	0.4966	0.040*	
C21	0.2744 (8)	0.9506 (10)	0.3966 (5)	0.029 (2)	
H21	0.3273	1.0021	0.3904	0.034*	
C22	0.1266 (8)	0.8491 (10)	0.3534 (5)	0.027 (2)	
H22	0.0740	0.8333	0.3165	0.032*	
S1	0.03282 (18)	0.8243 (2)	0.12350 (13)	0.0236 (5)	
O1	0.1186 (5)	0.7565 (7)	0.1159 (4)	0.0321 (17)	
O2	0.0432 (6)	0.8922 (7)	0.1865 (4)	0.0351 (18)	
O3	−0.0157 (6)	0.8948 (7)	0.0660 (4)	0.0353 (18)	
C23	−0.0529 (8)	0.6965 (11)	0.1288 (6)	0.033 (3)	
F1	−0.1388 (5)	0.7438 (7)	0.1347 (4)	0.0483 (19)	
F2	−0.0687 (6)	0.6231 (7)	0.0747 (4)	0.0506 (19)	
F3	−0.0193 (5)	0.6218 (7)	0.1815 (4)	0.0451 (18)	
S2	0.4912 (2)	0.6737 (3)	0.15718 (15)	0.0355 (7)	
O4	0.5019 (6)	0.7902 (9)	0.1973 (4)	0.043 (2)	
O5	0.3907 (6)	0.6430 (10)	0.1279 (5)	0.053 (2)	
O6	0.5539 (7)	0.5725 (8)	0.1845 (4)	0.046 (2)	
C24	0.5353 (10)	0.7271 (13)	0.0856 (7)	0.046 (3)	
F4	0.6289 (5)	0.7586 (8)	0.1038 (4)	0.056 (2)	
F5	0.4872 (7)	0.8264 (8)	0.0557 (4)	0.067 (3)	
F6	0.5285 (6)	0.6260 (8)	0.0427 (4)	0.055 (2)	

### Atomic displacement parameters (Å^2^)

**Table:**

	*U*^11^	*U*^22^	*U*^33^	*U*^12^	*U*^13^	*U*^23^
Ag1	0.0285 (4)	0.0302 (5)	0.0323 (5)	0.0106 (3)	0.0116 (3)	0.0109 (3)
Ag2	0.0345 (5)	0.0296 (5)	0.0286 (5)	0.0019 (3)	0.0124 (3)	0.0066 (3)
N1	0.028 (5)	0.018 (4)	0.032 (5)	0.009 (4)	0.010 (4)	0.006 (4)
N2	0.024 (5)	0.033 (5)	0.030 (5)	0.002 (4)	0.010 (4)	−0.003 (4)
N3	0.042 (6)	0.049 (6)	0.020 (5)	0.014 (5)	0.006 (4)	0.017 (4)
N4	0.033 (5)	0.024 (4)	0.027 (5)	−0.002 (4)	0.014 (4)	−0.001 (4)
N5	0.019 (4)	0.031 (5)	0.036 (5)	−0.001 (4)	0.006 (4)	0.001 (4)
N6	0.034 (5)	0.024 (5)	0.035 (5)	−0.011 (4)	0.005 (4)	−0.001 (4)
C1	0.031 (7)	0.047 (8)	0.052 (8)	0.006 (6)	0.002 (6)	0.005 (6)
C2	0.038 (7)	0.034 (7)	0.055 (8)	0.001 (5)	0.018 (6)	0.005 (6)
C3	0.037 (7)	0.052 (8)	0.054 (8)	0.007 (6)	0.024 (6)	0.022 (7)
C4	0.047 (7)	0.039 (7)	0.039 (7)	0.026 (6)	0.011 (6)	0.017 (6)
C5	0.027 (5)	0.027 (6)	0.034 (6)	0.014 (4)	0.015 (5)	0.001 (4)
C6	0.046 (7)	0.040 (7)	0.037 (7)	0.015 (6)	0.005 (6)	−0.004 (5)
C7	0.026 (5)	0.029 (6)	0.026 (5)	0.002 (4)	0.008 (4)	0.003 (4)
C8	0.033 (6)	0.025 (5)	0.035 (6)	−0.009 (5)	0.015 (5)	0.001 (5)
C9	0.029 (6)	0.041 (6)	0.022 (5)	−0.004 (5)	0.004 (4)	−0.001 (5)
C10	0.025 (5)	0.037 (6)	0.024 (5)	0.002 (5)	0.008 (4)	0.003 (5)
C11	0.027 (6)	0.034 (6)	0.030 (6)	−0.018 (5)	0.008 (5)	0.001 (5)
C12	0.042 (6)	0.024 (5)	0.035 (6)	0.003 (5)	0.026 (5)	0.010 (5)
C13	0.054 (8)	0.028 (6)	0.061 (8)	−0.001 (6)	0.037 (7)	−0.004 (6)
C14	0.066 (9)	0.024 (6)	0.047 (7)	−0.017 (6)	0.034 (7)	−0.011 (5)
C15	0.048 (7)	0.017 (5)	0.029 (6)	−0.017 (5)	0.020 (5)	−0.010 (4)
C16	0.032 (6)	0.027 (6)	0.036 (6)	−0.004 (5)	0.014 (5)	0.001 (5)
C17	0.039 (5)	0.029 (5)	0.036 (5)	−0.015 (4)	0.008 (4)	0.010 (4)
C18	0.025 (5)	0.009 (4)	0.035 (6)	0.000 (4)	0.003 (4)	0.000 (4)
C19	0.043 (6)	0.021 (5)	0.020 (5)	0.001 (5)	0.006 (5)	0.004 (4)
C20	0.039 (6)	0.026 (6)	0.034 (6)	−0.004 (5)	0.006 (5)	−0.002 (5)
C21	0.024 (5)	0.031 (6)	0.031 (6)	0.001 (4)	0.008 (5)	0.004 (5)
C22	0.033 (6)	0.021 (5)	0.027 (5)	−0.003 (4)	0.008 (5)	−0.003 (4)
S1	0.0243 (12)	0.0202 (12)	0.0250 (13)	−0.0021 (10)	0.0031 (10)	0.0036 (10)
O1	0.024 (4)	0.036 (4)	0.039 (4)	0.004 (3)	0.014 (3)	0.006 (4)
O2	0.044 (5)	0.029 (4)	0.033 (4)	−0.009 (4)	0.009 (4)	−0.006 (3)
O3	0.042 (5)	0.027 (4)	0.035 (4)	0.004 (3)	0.005 (4)	0.013 (3)
C23	0.029 (6)	0.026 (6)	0.043 (7)	−0.004 (5)	0.009 (5)	0.002 (5)
F1	0.022 (3)	0.051 (4)	0.074 (5)	−0.002 (3)	0.016 (3)	0.013 (4)
F2	0.065 (5)	0.036 (4)	0.049 (4)	−0.012 (4)	0.008 (4)	−0.020 (3)
F3	0.039 (4)	0.034 (4)	0.061 (5)	−0.001 (3)	0.008 (3)	0.026 (3)
S2	0.0376 (16)	0.0334 (15)	0.0401 (16)	0.0023 (12)	0.0185 (13)	0.0078 (12)
O4	0.039 (5)	0.052 (5)	0.039 (5)	0.014 (4)	0.013 (4)	−0.014 (4)
O5	0.036 (5)	0.060 (6)	0.068 (7)	−0.017 (4)	0.020 (5)	−0.007 (5)
O6	0.078 (7)	0.029 (4)	0.034 (5)	0.014 (4)	0.017 (4)	0.005 (4)
C24	0.050 (8)	0.045 (8)	0.044 (7)	0.006 (6)	0.015 (6)	0.009 (6)
F4	0.046 (4)	0.057 (5)	0.077 (6)	−0.020 (4)	0.039 (4)	−0.005 (4)
F5	0.090 (7)	0.061 (5)	0.054 (5)	0.032 (5)	0.029 (5)	0.038 (4)
F6	0.070 (5)	0.056 (5)	0.041 (4)	0.014 (4)	0.016 (4)	−0.016 (4)

### Geometric parameters (Å, º)

**Table:**

Ag1—N2^i^	2.151 (9)	C9—H9	0.9500
Ag1—N1	2.164 (9)	C10—H10	0.9500
Ag1—Ag2	3.0897 (12)	C11—H11	0.9500
Ag2—N4^ii^	2.151 (9)	C12—C13	1.378 (17)
Ag2—N5	2.169 (9)	C12—H12	0.9500
N1—C1	1.286 (16)	C13—C14	1.36 (2)
N1—C5	1.355 (14)	C13—H13	0.9500
N2—C10	1.336 (14)	C14—C15	1.362 (18)
N2—C11	1.357 (15)	C14—H14	0.9500
N2—Ag1^iii^	2.151 (9)	C15—C16	1.399 (15)
N3—C5	1.354 (15)	C15—H15	0.9500
N3—C6	1.458 (15)	C17—C18	1.541 (14)
N3—H3	0.8800	C17—H17A	0.9900
N4—C16	1.348 (14)	C17—H17B	0.9900
N4—C12	1.363 (14)	C18—C19	1.376 (15)
N4—Ag2^iv^	2.151 (9)	C18—C22	1.378 (15)
N5—C21	1.325 (14)	C19—C20	1.382 (16)
N5—C22	1.364 (14)	C19—H19	0.9500
N6—C16	1.371 (15)	C20—C21	1.401 (16)
N6—C17	1.437 (14)	C20—H20	0.9500
N6—H6	0.8800	C21—H21	0.9500
C1—C2	1.343 (19)	C22—H22	0.9500
C1—H1	0.9500	S1—O3	1.432 (8)
C2—C3	1.380 (19)	S1—O1	1.439 (8)
C2—H2	0.9500	S1—O2	1.459 (8)
C3—C4	1.385 (19)	S1—C23	1.817 (11)
C3—H3A	0.9500	C23—F2	1.331 (14)
C4—C5	1.406 (16)	C23—F3	1.335 (13)
C4—H4	0.9500	C23—F1	1.338 (13)
C6—C7	1.532 (15)	S2—O6	1.408 (9)
C6—H6A	0.9900	S2—O5	1.445 (9)
C6—H6B	0.9900	S2—O4	1.457 (9)
C7—C8	1.370 (15)	S2—C24	1.815 (13)
C7—C11	1.388 (16)	C24—F5	1.312 (15)
C8—C9	1.365 (16)	C24—F4	1.329 (15)
C8—H8	0.9500	C24—F6	1.365 (16)
C9—C10	1.374 (16)		
			
N2^i^—Ag1—N1	173.2 (3)	N4—C12—C13	122.1 (12)
N2^i^—Ag1—Ag2	82.3 (2)	N4—C12—H12	118.9
N1—Ag1—Ag2	91.7 (2)	C13—C12—H12	118.9
N4^ii^—Ag2—N5	157.1 (3)	C14—C13—C12	118.1 (13)
N4^ii^—Ag2—Ag1	106.3 (2)	C14—C13—H13	120.9
N5—Ag2—Ag1	92.4 (2)	C12—C13—H13	120.9
C1—N1—C5	117.0 (10)	C13—C14—C15	122.2 (11)
C1—N1—Ag1	121.4 (8)	C13—C14—H14	118.9
C5—N1—Ag1	121.4 (7)	C15—C14—H14	118.9
C10—N2—C11	117.8 (9)	C14—C15—C16	117.3 (11)
C10—N2—Ag1^iii^	119.9 (7)	C14—C15—H15	121.3
C11—N2—Ag1^iii^	122.0 (7)	C16—C15—H15	121.3
C5—N3—C6	123.5 (10)	N4—C16—N6	115.2 (9)
C5—N3—H3	118.3	N4—C16—C15	122.1 (10)
C6—N3—H3	118.3	N6—C16—C15	122.6 (10)
C16—N4—C12	118.0 (9)	N6—C17—C18	114.8 (9)
C16—N4—Ag2^iv^	127.9 (7)	N6—C17—H17A	108.6
C12—N4—Ag2^iv^	114.0 (7)	C18—C17—H17A	108.6
C21—N5—C22	119.9 (9)	N6—C17—H17B	108.6
C21—N5—Ag2	117.9 (7)	C18—C17—H17B	108.6
C22—N5—Ag2	121.3 (7)	H17A—C17—H17B	107.5
C16—N6—C17	122.3 (10)	C19—C18—C22	119.0 (10)
C16—N6—H6	118.9	C19—C18—C17	119.8 (9)
C17—N6—H6	118.9	C22—C18—C17	121.2 (9)
N1—C1—C2	129.9 (13)	C18—C19—C20	119.5 (10)
N1—C1—H1	115.1	C18—C19—H19	120.2
C2—C1—H1	115.1	C20—C19—H19	120.2
C1—C2—C3	114.1 (12)	C19—C20—C21	119.1 (10)
C1—C2—H2	123.0	C19—C20—H20	120.4
C3—C2—H2	123.0	C21—C20—H20	120.4
C2—C3—C4	120.6 (12)	N5—C21—C20	121.0 (10)
C2—C3—H3A	119.7	N5—C21—H21	119.5
C4—C3—H3A	119.7	C20—C21—H21	119.5
C3—C4—C5	119.0 (11)	N5—C22—C18	121.4 (10)
C3—C4—H4	120.5	N5—C22—H22	119.3
C5—C4—H4	120.5	C18—C22—H22	119.3
N3—C5—N1	117.8 (9)	O3—S1—O1	114.7 (5)
N3—C5—C4	122.8 (10)	O3—S1—O2	115.2 (5)
N1—C5—C4	119.4 (11)	O1—S1—O2	114.6 (5)
N3—C6—C7	114.7 (10)	O3—S1—C23	103.0 (5)
N3—C6—H6A	108.6	O1—S1—C23	103.6 (5)
C7—C6—H6A	108.6	O2—S1—C23	103.6 (5)
N3—C6—H6B	108.6	F2—C23—F3	108.3 (9)
C7—C6—H6B	108.6	F2—C23—F1	107.7 (9)
H6A—C6—H6B	107.6	F3—C23—F1	107.5 (9)
C8—C7—C11	116.8 (10)	F2—C23—S1	110.9 (8)
C8—C7—C6	119.6 (10)	F3—C23—S1	110.9 (8)
C11—C7—C6	123.5 (10)	F1—C23—S1	111.3 (8)
C9—C8—C7	121.8 (11)	O6—S2—O5	118.1 (6)
C9—C8—H8	119.1	O6—S2—O4	114.7 (5)
C7—C8—H8	119.1	O5—S2—O4	112.9 (6)
C8—C9—C10	117.9 (10)	O6—S2—C24	104.9 (6)
C8—C9—H9	121.1	O5—S2—C24	102.1 (6)
C10—C9—H9	121.1	O4—S2—C24	101.5 (6)
N2—C10—C9	123.0 (10)	F5—C24—F4	108.3 (11)
N2—C10—H10	118.5	F5—C24—F6	110.3 (11)
C9—C10—H10	118.5	F4—C24—F6	107.1 (11)
N2—C11—C7	122.7 (10)	F5—C24—S2	112.9 (9)
N2—C11—H11	118.7	F4—C24—S2	110.2 (9)
C7—C11—H11	118.7	F6—C24—S2	107.8 (9)
			
N2^i^—Ag1—Ag2—N4^ii^	0.5 (3)	C12—C13—C14—C15	−1.5 (19)
N1—Ag1—Ag2—N4^ii^	177.3 (3)	C13—C14—C15—C16	0.4 (17)
N2^i^—Ag1—Ag2—N5	−166.0 (3)	C12—N4—C16—N6	−179.8 (9)
N1—Ag1—Ag2—N5	10.9 (3)	Ag2^iv^—N4—C16—N6	−2.3 (14)
N2^i^—Ag1—N1—C1	110 (3)	C12—N4—C16—C15	−1.6 (15)
Ag2—Ag1—N1—C1	82.1 (9)	Ag2^iv^—N4—C16—C15	175.8 (8)
N2^i^—Ag1—N1—C5	−67 (3)	C17—N6—C16—N4	−164.2 (9)
Ag2—Ag1—N1—C5	−94.4 (7)	C17—N6—C16—C15	17.7 (16)
N4^ii^—Ag2—N5—C21	−51.1 (13)	C14—C15—C16—N4	1.2 (16)
Ag1—Ag2—N5—C21	93.7 (8)	C14—C15—C16—N6	179.2 (10)
N4^ii^—Ag2—N5—C22	118.2 (10)	C16—N6—C17—C18	−89.4 (13)
Ag1—Ag2—N5—C22	−96.9 (8)	N6—C17—C18—C19	176.1 (10)
C5—N1—C1—C2	1 (2)	N6—C17—C18—C22	−5.8 (16)
Ag1—N1—C1—C2	−175.3 (12)	C22—C18—C19—C20	−2.8 (15)
N1—C1—C2—C3	−1 (2)	C17—C18—C19—C20	175.3 (10)
C1—C2—C3—C4	0.9 (19)	C18—C19—C20—C21	−0.2 (16)
C2—C3—C4—C5	−0.3 (19)	C22—N5—C21—C20	−2.4 (16)
C6—N3—C5—N1	164.4 (10)	Ag2—N5—C21—C20	167.1 (8)
C6—N3—C5—C4	−16.9 (17)	C19—C20—C21—N5	2.8 (17)
C1—N1—C5—N3	178.2 (11)	C21—N5—C22—C18	−0.7 (15)
Ag1—N1—C5—N3	−5.1 (13)	Ag2—N5—C22—C18	−169.8 (8)
C1—N1—C5—C4	−0.5 (15)	C19—C18—C22—N5	3.3 (15)
Ag1—N1—C5—C4	176.2 (8)	C17—C18—C22—N5	−174.8 (10)
C3—C4—C5—N3	−178.6 (11)	O3—S1—C23—F2	61.0 (9)
C3—C4—C5—N1	0.0 (17)	O1—S1—C23—F2	−58.8 (9)
C5—N3—C6—C7	95.1 (14)	O2—S1—C23—F2	−178.7 (8)
N3—C6—C7—C8	170.4 (10)	O3—S1—C23—F3	−178.7 (8)
N3—C6—C7—C11	−6.0 (17)	O1—S1—C23—F3	61.6 (9)
C11—C7—C8—C9	−1.6 (16)	O2—S1—C23—F3	−58.3 (9)
C6—C7—C8—C9	−178.3 (11)	O3—S1—C23—F1	−59.0 (9)
C7—C8—C9—C10	1.4 (17)	O1—S1—C23—F1	−178.7 (8)
C11—N2—C10—C9	2.0 (16)	O2—S1—C23—F1	61.3 (9)
Ag1^iii^—N2—C10—C9	175.4 (8)	O6—S2—C24—F5	−177.0 (10)
C8—C9—C10—N2	−1.6 (17)	O5—S2—C24—F5	59.3 (11)
C10—N2—C11—C7	−2.3 (15)	O4—S2—C24—F5	−57.4 (11)
Ag1^iii^—N2—C11—C7	−175.5 (8)	O6—S2—C24—F4	−55.7 (11)
C8—C7—C11—N2	2.1 (16)	O5—S2—C24—F4	−179.3 (9)
C6—C7—C11—N2	178.7 (11)	O4—S2—C24—F4	64.0 (10)
C16—N4—C12—C13	0.5 (15)	O6—S2—C24—F6	60.9 (10)
Ag2^iv^—N4—C12—C13	−177.3 (9)	O5—S2—C24—F6	−62.8 (10)
N4—C12—C13—C14	1.1 (17)	O4—S2—C24—F6	−179.5 (8)

Symmetry codes: (i) −*x*+1, *y*+1/2, −*z*+1/2; (ii) −*x*, *y*+1/2, −*z*+1/2; (iii) −*x*+1, *y*−1/2, −*z*+1/2; (iv) −*x*, *y*−1/2, −*z*+1/2.

### Hydrogen-bond geometry (Å, º)

**Table:**

*D*—H···*A*	*D*—H	H···*A*	*D*···*A*	*D*—H···*A*
N3—H3···O4	0.88	2.20	3.021 (12)	156
N6—H6···O2^iv^	0.88	2.42	3.159 (12)	142
C1—H1···O1	0.95	2.56	3.389 (16)	146
C6—H6*A*···F6^v^	0.99	2.55	3.282 (15)	131
C9—H9···O3^vi^	0.95	2.44	3.329 (14)	156
C10—H10···O1^iii^	0.95	2.57	3.373 (14)	142
C12—H12···O4^vii^	0.95	2.51	3.331 (15)	145
C17—H17*A*···O3^iv^	0.99	2.42	3.186 (14)	134
C21—H21···F6^i^	0.95	2.54	3.325 (14)	140

Symmetry codes: (i) −*x*+1, *y*+1/2, −*z*+1/2; (iii) −*x*+1, *y*−1/2, −*z*+1/2; (iv) −*x*, *y*−1/2, −*z*+1/2; (v) *x*, −*y*+3/2, *z*+1/2; (vi) *x*+1, −*y*+3/2, *z*+1/2; (vii) *x*−1, *y*, *z*.
